# Potential Use of Interleukin-10 Blockade as a Therapeutic Strategy in Human Cutaneous Leishmaniasis

**DOI:** 10.1155/2015/152741

**Published:** 2015-10-01

**Authors:** Lucio Roberto Castellano, Laurent Argiro, Helia Dessein, Alain Dessein, Marcos Vinícius da Silva, Dalmo Correia, Virmondes Rodrigues

**Affiliations:** ^1^Laboratory of Immunology, Federal University of Triângulo Mineiro, 38025-180 Uberaba, MG, Brazil; ^2^Human Immunology Research and Education Group-GEPIH, Technical Health School, Federal University of Paraíba, 58051-900 João Pessoa, PB, Brazil; ^3^Laboratory of Parasitology Mycology, INSERM U399, Faculty of Medicine, 27 boulevard Jean Moulin, 13385 Marseille Cedex 05, France; ^4^Infectious Diseases Division, Federal University of Triângulo Mineiro, 38025-180 Uberaba, MG, Brazil

## Abstract

Interleukin-10 overproduction has been associated with worse prognosis in human cutaneous leishmaniasis, while IFN-*γ*-dependent responses are associated with parasite killing and host protection. Innovative strategies are needed to overcome therapeutic failure observed in endemic areas. The use of monoclonal antibody-based immunotherapy targeting IL-10 cytokine was evaluated here. Partial IL-10 blockade in *Leishmania braziliensis* whole soluble antigen-stimulated cells from endemic area CL patients with active or healed lesions and asymptomatic controls was evaluated. Overall decrease in IL-10, IL-4, and TNF-*α* production was observed in all groups of subjects. Only patients with active lesions still produced some levels of TNF-*α* after anti-IL-10 stimulation in association with *Leishmania* antigens. Moreover, this strategy showed limited modulatory effects on IFN-*γ*-dependent chemokine CXCL10 production. Results suggest the potential immunotherapeutic use of partial IL-10 blockade in localized cutaneous leishmaniasis.

## 1. Background

Infections due to the protozoa from the genus* Leishmania* still constitute a major public health problem worldwide. Patients suffer from all clinical forms of the disease, without a specific vaccine or a safe and effective treatment. Interleukin-10 was implicated on T cell unresponsiveness observed in visceral leishmaniasis (VL) patients infected with* L. donovani* [1]. Cutaneous leishmaniasis (CL) is believed to present an unbalanced Th1/Th2 response during its acute phase with clinical resolution being an IFN-*γ*-dependent event, whereas lesion progression and therapeutic failure are related to IL-10 overproduction [2–6]. The reduction of IL-10 levels using neutralizing anti-IL-10 or anti-IL-10R monoclonal antibodies (mAb) might be useful as immunotherapeutic adjuvants immunotherapies to prevent or treat experimental infections by many pathogens by favoring the production of IFN-*γ* and TNF-*α* [7]. Anti-IL-10 mAbs when added to cell cultures restored the proliferative response of peripheral blood mononuclear cells (PBMC) from a VL patient [1] and increased the IFN-*γ* production by CD4^+^CD25^−^ T cells cocultured with intralesional Treg cells of* L. guyanensis* infected CL patients [2]. Furthermore, PBMC from unexposed subjects showed an increase on IFN-*γ*, TGF-*β*, and reactive nitrogen production when cultured in presence of* Leishmania* antigens and anti-IL-10 mAb [8]. All these data suggest that new CL vaccines and therapies should involve an IL-10-neutralizing strategy. Considering that IFN-*γ*-dependent responses are essential anti-*Leishmania* defense mechanisms and that their magnitude is modulated by IL-10, the evaluation of any product of the IFN-*γ* signaling cascade, such as CXCL10, rather than the cytokine alone, would improve data interpretation of how successful this network is modulated in CL patients. Our results suggest that partial IL-10 neutralization using anti-hIL-10 mAb is able to reduce Th2 profile and increase protective IFN-*γ*-related response in peripheral PBMC from subjects living in areas where* Leishmania braziliensis* (Lb) is endemic.

## 2. Findings

### 2.1. Materials and Methods

#### 2.1.1. Study Population

For this study, 18 male individuals were selected from a previously characterized CL endemic area located in Buerarema Village, Bahia State, Brazil [6]. The groups consisted of 6 patients with active lesions (aCL), 6 patients with chemotherapeutically healed lesions (hCL), and 6 asymptomatic uninfected endemic area subjects (asymptomatic). The mean age of these individuals was 33, 39, and 35 years, respectively. The evolution time of the lesions in the aCL group was between 1 and 2 months, while hCL group presented healed lesions with more than 1 year. All individuals, including asymptomatic ones, lived for at least 22 years in the area, without any migratory event within this period. The aCL and hCL patients were treated with meglumine antimoniate following Brazilian Ministry of Health procedures, as previously described [4].

#### 2.1.2. Mononuclear Cells Isolation and Culture

Peripheral blood mononuclear cells (PBMC) were isolated by Ficoll-Paque centrifugation (Pharmacia, Uppsala, Sweden), at 400 ×g, 20 min at room temperature, washed three times in RPMI medium (Gibco, Grand Island, NY), and suspended in DMEM medium (Gibco), supplemented with 50 *μ*M 2-mercaptoethanol, 2 mM L-glutamine (Gibco), 40 *μ*g/mL gentamicin, and 5% fetal calf serum (Gibco). Cell cultures (2 × 10^6^ cells/well) were stimulated with 5 *μ*g/mL whole soluble Lb antigens in presence or not of 10 *μ*g/mL of anti-human IL-10 monoclonal antibody (anti-hIL-10 mAb) (R&D Systems, MAB217, Clone 23738) for 24 h at 37°C, 5% CO_2_.

#### 2.1.3. Cytokine and Chemokine Detection by ELISA

Cytokines (IL-10, IL-4, and TNF-*α*) and CXCL10 levels were measured by ELISA using pair-matched antibodies (Pharmingen, San Diego, CA) as described [4]. Briefly, 96-well ELISA microplates (Nunc, Denmark) were sensitized overnight with 100 *μ*L of 1 *μ*g/mL specific monoclonal antibody (Mabtech, USA, and Pharmingen, USA). The plates were then washed with PBS 0.05% Tween-20 (Sigma) and incubated with 2% bovine serum albumin (BSA; Sigma) in PBS. Plates were incubated overnight with 100 *μ*L of 1 : 2 dilutions of culture supernatants in 2% BSA-PBS or with recombinant human cytokine (Pharmingen). Then, 1 *μ*g/mL of appropriate biotinylated anti-cytokine monoclonal antibody was added (Mabtech and Pharmingen) for 2 h at 37°C, followed by washing and incubation with alkaline phosphatase-conjugated streptavidin for 2 h at 37°C. Finally, enzymatic activity was developed by incubation with p-nitrophenyl phosphate (Sigma). Absorbance was read at 405 nm in a microplate reader (BioRad, USA).

#### 2.1.4. Statistical Analysis

Comparison of cytokines and chemokine production among groups of patients was performed by Kruskal-Wallis followed by Dunn's post hoc test. Statistical significance adopted *P* < 0.05. The equation used for data analysis was

(1) cytokine  detected  in  AgLb+aIL10  supernatantcy  AgLb  W−1 −cytokine  detected  in  AgLb  supernatant ·cytokine  detected  in  AgLb  supernatant−1×100.

## 3. Results

**Figure 1:**
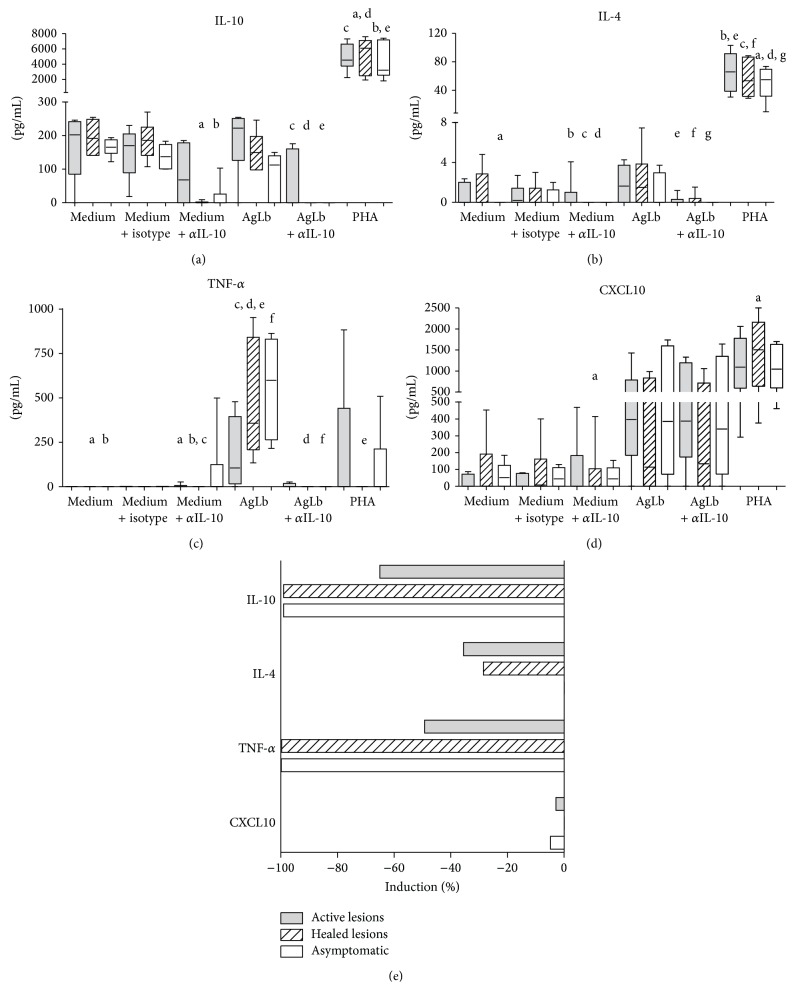
Modulatory effects of* in vitro* IL-10 blockade over T cell response in patients with cutaneous leishmaniasis. Cytokines IL-10, IL-4, TNF-*α*, and chemokine CXCL10 produced by PBMC were measured by ELISA. Cells were cultured for 24 h in the presence of 5 *μ*g/mL* L. braziliensis* antigens alone or in combination with 5 *μ*g/mL anti-human IL-10 mAb. Patients were grouped according to the presence of active lesions (*n* = 6), healed lesions (*n* = 6), or lack of any disease history (*n* = 6). (a)–(d) Levels of each cytokine production are plotted. The horizontal line represents the median, the bar 25th–75th percentiles, and the vertical line the 10th–90th percentiles. Equal letters mean Kruskal-Wallis test, *P* < 0.05, and post hoc Dunn test statistically significant. (e) Percentage of induction in the production of each cytokine and chemokine by IL-10 blockade was evaluated considering {[(AgLb + *α*IL-10) − AgLb]/AgLb}*∗*100. Bars indicate median inhibition values for each group. ^*∗*^ *P* < 0.05.

The potential use of anti-IL-10 blocking mAb was tested in PBMC from individuals living in an endemic area of cutaneous leishmaniasis. As shown in Figure 1, there was only a partial blockade of the anti-IL-10 mAb used indicated by the remaining IL-10 detection in all groups. Decreased IL-4 levels were observed in cultures from all studied subjects, while decreased TNF-*α* production was observed in healed and asymptomatic individuals only. Patients with active lesions, however, presented a concomitant increase in TNF-*α* and in CXCL10 after IL-10 blockade.

The percentage of inhibition of cytokine production in Lb-stimulated PBMC cocultured in the presence of anti-IL-10 mAb was also evaluated (Figure 1(e)). Interestingly, anti-IL-10 mAb induced an overall decrease of IL-10, IL-4, TNF-*α*, and to a lesser extent CXCL10 production by cells from all subjects. Patients with active lesions were less affected by the IL-10 blockade, showing increased capacity of cytokine production in this condition. Moreover, CXCL10 production by healed patients and IL-4 production by cells from asymptomatic individuals showed not to be modulated by IL-10 blockade.

## 4. Discussion

Strategies focusing on the control of the cytokine milieu are really important in combating many infectious and noninfectious diseases. One of the most immunomodulatory cytokines to be elected for such control is IL-10. It is produced by different cell types with effects that vary from the activation to the downregulation of many cell types depending on cellular conditions and activation status [7]. One important aspect of the IL-10 biology is that this cytokine is able to modulate immunological responses of both Th1 and Th2 patterns. Effective cellular immune* response* and clearance of* Leishmania* infection in humans have shown to be dependent on Th1 cytokines like TNF-*α* and IFN-*γ*, while parasite persistence and disease establishment are favored by IL-4 and IL-10 overproduction [1–6]. It is noteworthy that the control of the IL-10 production would bring new insights to the therapy of both cutaneous and visceral forms of the disease especially in those cases where parasites present drug resistance or when patients display low tolerance to therapy. However, few data is available on the effect of IL-10 neutralization in human leishmaniasis. In VL form of the disease, the administration of an anti-IL-10 mAb to PBMC cultures restored the unresponsiveness of T cell proliferation against* Leishmania* antigens in one patient [1]. More recently, a similar neutralization strategy in cultures of splenic aspirate cells from VL patients promoted a decrease in the number of amastigotes concomitantly with an increased production of IFN-*γ* and TNF-*α* [9]. Moreover, PBMC from unexposed subjects produced higher levels of IFN-*γ*, TGF-*β*, and reactive nitrogen species when cultured in the presence of* Leishmania* antigens and anti-IL-10 mAb [8]. In cutaneous leishmaniasis, the only existing data is a recent report on which the addition of an anti-IL-10 mAb abrogated the* in vitro* modulatory effect of intralesional CD4^+^CD25^+^Foxp3^+^ Treg cells and promoted an increase in IFN-*γ* production by effector T cells from* L. guyanensis* infected individuals [2]. Recent data suggested that human IFN-*γ*-producing CD4^+^ T cells seem to be essential for inducing parasite killing by macrophages,* in vitro*. These cells lost their activity after anti-IFN-*γ* mAb addition to the culture [10]. On the other hand, CD8^+^ T cells have been associated with tissue damage, local necrosis, and lesion progression in CL patients and infected mice [10, 11]. In both papers, the cytolytic activity of CD8^+^ T cells observed in CL patients seems not to be directed against parasite killing but to tissue destruction. Inhibition of IFN-*γ* in the cell cultures did not modulate the cytolytic activity of CD8^+^ T cells but increased the infection index of cocultured macrophages infected with* L. braziliensis*. These data suggest that CD4^+^ T cells are the main sources of anti-*Leishmania* IFN-*γ*-dependent protective immune responses, which indicates that increasing the activity of Th1 cell population would function as an important strategy in CL therapy. The importance of IL-10 in modulating the cytolytic activity of CD8^+^ T cells in human leishmaniasis is unclear.

Interestingly, our data showing the partial neutralization of the IL-10 production in PBMC was divergent among habitants from the same area where CL is endemic. Infected individuals were still able to respond to the IL-10 neutralization by producing near to basal levels of cytokines as observed in the anti-*Leishmania* T cell response. Decreased CXCL10 modulation observed here indicates that IFN-*γ*-dependent responses could be restored in these individuals and would ensure disease recovery, parasite control, and a better prognosis. In contrast, there was a limited downmodulation on TNF-*α* production in aCL group in response to anti-IL-10 mAb. This result would be considered as a drawback of the potential therapeutic administration of anti-IL-10 mAbs to CL patients. Strong evidence suggests that excessive proinflammatory responses, especially those mediated by TNF-*α*, lead to tissue damage and mucosal commitment in American tegumentary leishmaniasis [12–15]. In this context, the adoption of therapeutic strategies aiming at the complete depletion of IL-10-dependent response should not be the best option for treating CL patients. For that reason, a partial blockade of IL-10 was adopted in this study. Overall decrease on cytokine production observed in uninfected individuals suggests that the adoption of this strategy is only effective on susceptible individuals whose immune response tends to be Th2-biased and modulated by IL-10 [4, 6]. Though limited to a small number of subjects, our work reinforces the idea that an unbalanced immunomodulatory response might be detrimental for successful CL treatment and lesion healing in* L. braziliensis* infection [4, 6, 13–16].

As discussed here, our data complements previous studies on IL-10 blockade strategy in human* Leishmania* infection. Considering the host-parasite interplay, independently on the clinical form of the disease, a partial blockade of the IL-10 would favour parasite clearance, lesion healing, and the establishment of an effective anti-*Leishmania* immune response. In human CL, our work is the first to adopt this strategy in* L. braziliensis* infection, which complements the previous data on IL-10 blockade in* L. guyanensis* infection [2].

Traditionally, it has been shown that VL patients treated with a combination of recombinant human INF-*γ* and pentavalent antimony had an increased successful rate and better disease recovery [17, 18]. In CL, the immunotherapeutic approaches already adopted were based mostly on the administration of killed or pasteurized* Leishmania* parasites alone or in addition to BCG showing elevated treatment efficacy and lesion healing [19]. One work adopted the administration of GM-CSF to a small number of patients showing a 100% cure of CL lesions [20]. Collectively, these data suggest that studies about new potential immunotherapies should be performed for both VL and CL treatments.

Here, we explore, for the first time, the potential role of IL-10 blockade in humans infected with* L. braziliensis* and bring some perspectives on the generation of new immunotherapies for cutaneous leishmaniasis.

## Abbreviations

aCL: Active cutaneous leishmaniasis lesions
AgLb: * Leishmania braziliensis* protein extract
CL: Cutaneous leishmaniasis
hCL: Healed cutaneous leishmaniasis lesions
Lb: * Leishmania braziliensis*
mAb: Monoclonal antibody
Treg: T regulatory lymphocytes
VL: Visceral leishmaniasis.
